# Reconstructing microbial communities

**DOI:** 10.1186/gb-2011-12-s1-i13

**Published:** 2011-09-19

**Authors:** Mihai Pop

**Affiliations:** 1 University of Maryland, College Park, MD, USA

## 

Metagenomic studies have allowed an unprecedented view of the microbial communities that inhabit our world and our bodies. Deep sequencing data have already been generated from several environments, as well as from various human body sites. As more data are generated, we are beginning to understand the structure of our commensal microbial communities and how microbes affect our health. Analyzing the metagenomic data, however, poses significant computational challenges, because few software tools are available that can handle the volume and characteristics of the data being generated. In my talk, I will primarily focus on the challenges posed by metagenomic assembly and will outline recent research in my laboratory aimed at meeting these challenges. I will also describe some of the analyses that can be performed on the assembled data but would not be possible in read-based analyses.

